# On the Proportionality Between Area and Load in Line Contacts

**DOI:** 10.1007/s11249-018-1061-7

**Published:** 2018-08-02

**Authors:** J. S. van Dokkum, M. Khajeh Salehani, N. Irani, L. Nicola

**Affiliations:** 1 Department of Materials Science and Engineering, Delft University of Technology, 2628 CD Delft, The Netherlands; 2 Department of Industrial Engineering, University of Padova, 35131 Padua, Italy

**Keywords:** Root-mean-square gradient, Random rough surface, Contact area, Reduced pressure, Greenwood and Williamson

## Abstract

The relative contact area of rough surface contacts is known to increase linearly with reduced pressure, with proportionality factor $\kappa$. In its common definition, the reduced pressure contains the root-mean-square gradient (RMSG) of the surface. Although easy to measure, the RMSG of the entire surface does not coincide, at small loads, with the RMSG over the actual contact area ${\bar{g}}_{\text {r}}$, which gives a better description of the contact between rough surfaces. It was recently shown that, for Hertzian contacts, linearity between area and load is indeed obtained only if the RMSG is determined over the actual contact area. Similar to surface contacts, in line contacts, numerical data are often studied using theories that predict linearity by design. In this work, we revisit line contact problems and examine whether or not the assumption of linearity for line contacts holds true. We demonstrate, using Green’s function molecular dynamics simulations, that $\kappa$ for line contacts is not a constant: It depends on both the reduced pressure and the Hurst exponent. However, linearity holds when the RMSG is measured over the actual contact area. In that case, we could compare $\kappa$ for line and surface contacts and found that their ratio is approximately 0.9. Finally, by analytically deriving the proportionality factor using ${\bar{g}}_{\text {r}}$ in the original model of Greenwood and Williamson, a value is obtained that is surprisingly in good agreement with our numerical results for rough surface contacts.

## Introduction

It is well established that for the elastic contact of random rough surfaces, the equation

1 $$\begin{aligned} a_{\text {rel}} = \kappa \,p^{*} \end{aligned}$$

provides a good description of the relation between the relative contact area $a_{\text {rel}}$ and the reduced pressure $p^{*}$ [1–8]. Here, $a_{\text {rel}}$ is defined as the ratio of the actual contact area $a_\text {act}$ (the area over which the gap between the two solids is zero) to the nominal contact area $a_\text {nom}$. Besides, $p^{*}\equiv p/(\bar{g}\,E^{*})$, where $E^{*}$ is the contact modulus; *p* is the nominal contact pressure, and $\bar{g}$ is the root-mean-square gradient (RMSG) calculated over the nominal contact area. The linear relation in Eq. (1) holds true when the infinitesimal contact condition is assumed, i.e., $p^{*}$ is small compared to 1. For surfaces with random roughness, several authors [2, 3, 7] have found a proportionality factor $\kappa$ weakly dependent on the Hurst roughness exponent and slightly greater than 2.

Although the RMSG of the entire rough surface is easy to measure, it does not directly reflect the physics of the problem, given that it does not coincide with the RMSG over the actual contact area. It was recently shown by Müser [9] that Eq. (1) does not hold for 2D single smooth axisymmetric asperity contacts, unless one replaces $\bar{g}$ with the RMSG calculated over the actual contact area ${\bar{g}}_{\text {r}}$. In the case of random rough surface contacts, $\bar{g}$ and $\bar{g}_{\text {r}}$ are expected to be negligibly different, but it is unknown whether this is also the case for line contacts. Nonetheless, similar to surface contacts, also for line contacts, numerical data are often fitted to laws that enforce linearity by design, e.g., see the work by Scaraggi et al. [10]. Here, we intend to investigate, with Green’s function molecular dynamics simulations, to which extend the assumption of linearity for line contacts holds true. Also, we compute the proportionality factor $\kappa$ using both definitions of RMSG in line and surface contacts, with the aim of finding the scaling factor between $\kappa$ values for 1D and 2D contacts. In this analysis, besides random rough surfaces also single smooth asperities are considered.

Our interest in studying 1D contacts, which is shared by various authors [10–18], stems from the fact that they are computationally less costly than 2D contacts, and therefore more suitable to study contact problems that go beyond linear elasticity. Consequently, the results presented in this work can provide a means of comparison for future contact simulations that describe materials that behave inelastically, for instance materials that deform by dislocation plasticity [19]. Another reason for studying line contacts is that in many practical applications rough surfaces are strongly anisotropic as a result of machining and surface treatment, e.g., unidirectional polished surfaces [20].

The simulations in this work show that linearity between relative contact area and load for line contacts is found, only provided that the RMSG is calculated over the actual contact area. This result has inspired us to check the effect of using $\bar{g}_{\text {r}}$ when deriving the proportionality factor $\kappa _{\text {r}}$ in the classical Greenwood and Williamson (GW) model [21]. Despite the simplicity of the original GW model, which does not even include elastic interactions, the agreement between the analytically derived $\kappa _{\text {r}}$ and that obtained through random rough surface contact simulations is surprisingly in good agreement.

The numerical analysis is performed by applying the Green’s function molecular dynamics (GFMD) technique of Campa$\tilde{\text {n}}$á and Müser [3] to non-adhesive contacts between elastic solids. Throughout this work, the roughness is mapped on a rigid indenter and the substrate is a semi-infinite incompressible elastic solid with an initially flat surface.

## Calculation of $\kappa _{\text {r}}$ for Single Smooth Asperity Contacts

Before modeling rough surfaces, we start by showing that our numerical results capture the proportionality factor $\kappa _{\text {r}}$ for 1D and 2D single smooth axisymmetric asperity contacts. The analytical results for Hertzian contacts were provided by Müser [9]. Also in his study, the reduced pressure is defined as $p^*_{\text {r}} \equiv p/(\bar{g}_{\text {r}}\,E^{*})$, with ${\bar{g}}_{\text {r}}$ being the RMSG calculated over the actual contact area, while *p* is load divided by an arbitrary but fixed reference area. Here, we show for the first time that also for infinitely long smooth cylindrical indenters the linear relation of Eq. (1) holds if the RMSG is calculated over the actual contact area, instead of the nominal contact area. Let us consider a single infinitely long and smooth cylinder that indents a semi-infinite incompressible elastic solid. The parabolic approximation of the height profile of the indenter is given by

2 $$\begin{aligned} h(\rho )=\frac{R}{2}\left( \frac{\rho }{R}\right) ^{2}, \end{aligned}$$

where $\rho$ is the distance from the vertical axis of symmetry and *R* is the radius of the cylinder. We start by assuming that the relation

3 $$\begin{aligned} a_{\text {rel}}=\frac{\kappa _{\text {r}}\,p}{\bar{g}_{\text {r}}\,E^*}, \end{aligned}$$

is valid for the current contact problem. By defining *p* as the load *L* averaged over the nominal contact area $a_\text {nom}$, this equation can be rewritten as

4 $$\begin{aligned} 2\,{c}=\frac{\kappa _{\text {r}}\,L}{\bar{g}_{\text {r}}\,E^*}, \end{aligned}$$

where *c* is the half-width of the actual contact area $a_\text {act}$. It follows from [22] that

5 $$\begin{aligned} L=\frac{\pi E^*{c}^2 }{4R}. \end{aligned}$$

Furthermore, the RMSG determined over the actual contact area $\bar{g}_{\text {r}}$ is obtained as

6 $$\begin{aligned} \bar{g}_{\text {r}}=\sqrt{\frac{2\int _0^{{c}} \left( \frac{\partial h}{\partial \rho }\right) ^2 \, \mathrm{d}\rho }{2\,{c}}} = \frac{{c}}{R\,\sqrt{3}}. \end{aligned}$$

Substituting the relations for *L* and $\bar{g}_{\text {r}}$ in Eq. (4) gives

7 $$\begin{aligned} \kappa _{\text {r}}=\frac{8}{\pi \,\sqrt{3}} \simeq 1.47. \end{aligned}$$

Note that the obtained proportionality constant is smaller than that of the Hertzian contact (see Table 1 for a comparison between the parameters of Hertzian and cylindrical contacts), and the ratio is $\kappa ^{\text {1D}}_{\text {r}}/\kappa ^{\text {2D}}_{\text {r}}\simeq 0.88$.

**Table 1:** Cylindrical and Hertzian contact parameters

	${a_\text {act}}$	*L*	$\bar{g}_{\text {r}}$	$\kappa _{\text {r}}$
1D (cylindrical)	$2\,{c}$	$\pi E^*{c}^2 /(4R)$	${c}/\left(R\,\sqrt{3} \right)$	1.47
2D (Hertzian [9])	$\pi \,{c}^2$	$\sqrt{\pi }\,{{\Gamma}}(2) E^*{c}^3/({{\Gamma}}(2.5)R)$	${c}/\left(R\,\sqrt{2} \right)$	1.66

**Fig. 1:**
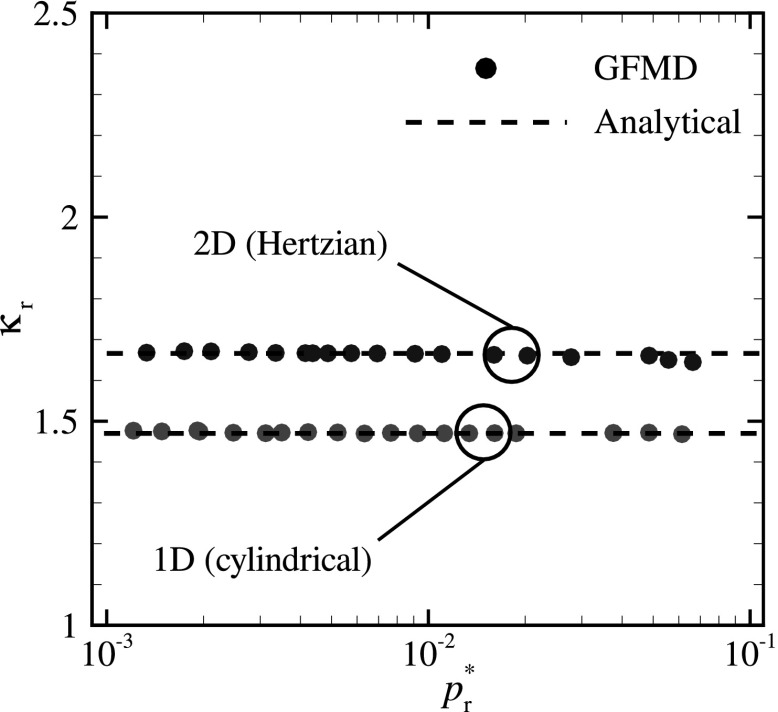
The numerical and analytical predictions of the proportionality factor $\kappa _{\text {r}}$ for smooth cylindrical and Hertzian indenters

The analytical results are used as a means of validation for our GFMD simulations, as shown in Fig. 1, where $\kappa _{\text {r}}$ is shown as a function of $p^{*}_{\text {r}}$ in the infinitesimal contact regime.

In GFMD, the surface of the elastic solid is first discretized with a number of equi-spaced grid points, which interact with each other through an effective stiffness [23]. Subsequently, the response of the material to the external loading is obtained using damped dynamics in Fourier space, by only considering the interactions of the surface grid points with their degrees of freedom coupled to the external load [7]. We note that in this work, through the periodicity of the discrete Fourier transforms [24], periodic boundary conditions are intrinsically enforced.

A minimum of $n=2^{13}$ equi-spaced grid points in each direction are employed to discretize the surfaces. Here, the ratio of the width of the periodic unit cell $\mathcal {L}$ to indenter radius *R* is set as $\mathcal {L}/R=4$. This guarantees that adjacent indenters do not interact within the selected pressure range.

For the numerical calculation of $\bar{g}_{\text {r}}$ the following procedure is adopted: If point *i* is in contact along the* x*- and/or the* y*-direction, the local mean-square gradient at point *i* is calculated as

8 $$\begin{aligned} g^2_i=\frac{1}{2}\left[ \left( \frac{h_i - h_{i+1}}{l}\right) ^2+ \left( \frac{h_i - h_{i-1}}{l}\right) ^2\right] , \end{aligned}$$

where $h_i$ is the height profile of the indenter at point *i* and *l* is the spacing between the grid points. Subsequently, the value of $\bar{g}^2_{\text {r}}$ is obtained as

9 $$\begin{aligned} \bar{g}^2_{\text {r}}=\frac{\sum \limits _{i=1}^{n_\text {act}} g^2_i}{n_\text {act}}, \end{aligned}$$

where $n_\text {act}$ is the total number of actual contact points, i.e., the points where the gap between the two solids is zero.

The agreement between the numerical and analytical results in Fig. 1 supports the validity of the numerical model. In the following, the same model is used to study random rough contacts.

## Random Rough Line Contacts

In the following, we assume that the indenter has a self-affine roughness with a Gaussian height distribution. The roughness is generated by means of the spectral method described in [25]. The power spectrum density function $C\left( q\right)$ of the self-affine roughness [26] is given by

10 $$\begin{aligned} {C\left( q\right) \equiv C\left( q_{\text {r}}\right) \times } {\left\{ \begin{array}{ll} {1}&{}\quad {\text {for}\, \ \lambda _{\text {r}}< \dfrac{2\pi }{q}\le \mathcal {L};} \\ {\left( \dfrac{q}{q_{\text {r}}}\right) ^{-(1+2H)}}&{}\quad {\text {for}}\, \ { \lambda _{\text {s,H}} < \dfrac{2\pi }{q} \le \lambda _{\text {r}} ;}\\ {0}&{}\quad {\text {for}}\, \ {\lambda _{s} \le \dfrac{2\pi }{q}\le \lambda _\text {s,H} ,} \end{array}\right. } \end{aligned}$$

where the fractal dimension is $D_\text {f}=2-H$, and $C\left( q_{\text {r}}\right)$ is scaled to obtain the desired RMSG $\bar{g}$ [27]. Here, $\lambda _{\text {r}}$ is the roll-off wavelength, $\mathcal {L}$ the longest wavelength and width of the periodic unit cell, $\lambda _\text {s,H}$ the roll-on wavelength, and $\lambda _\text {s}$ is the shortest wavelength. The value of ${\bar{g}}$ is taken to be constant and equal to 0.001. The roll-off wavelength is taken to be constant, $\lambda _{\text {r}} = 20\;\mu$m. Besides, $\varepsilon _\text {t} = \lambda _{\text {r}} / \mathcal {L}$ is set to 1 / 8 as according to [28] any $\varepsilon _\text {t} \leqslant 1/4$ provides an acceptable probability density of heights for rough surfaces. The roll-on wavelength $\lambda _\text {s,H}$ is selected such that $\varepsilon _\text {f} = \lambda _\text {s,H} / \lambda _{\text {r}} = 1/512$, similar to [10]. The continuum discretization $\varepsilon _\text {c} = \lambda _\text {s} / \lambda _\text {s,H}$ is set equal to 1 / 64 [25]. This assures numerical convergence for all cases studied here including low pressure values and all Hurst exponents, namely $H=0.2$, 0.5, and 0.8, as discussed in [10].

In order to account for the random nature of the roughness, GFMD calculations are performed for ten different randomly generated rough profiles for any given Hurst exponent. Thereafter, the statistical average is taken over the obtained results.

**Fig. 2:**
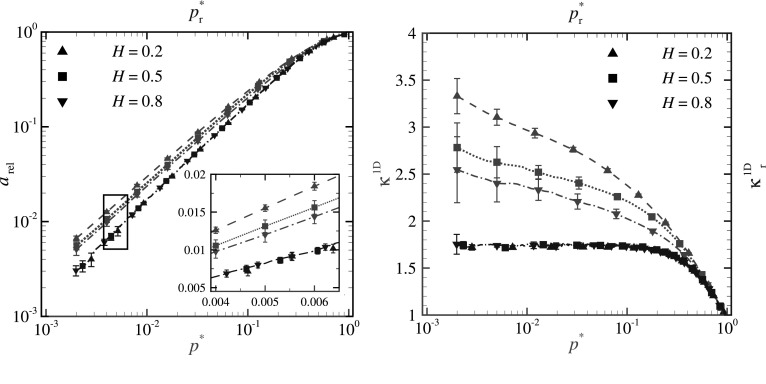
**a** The relative contact area $a_{\text {rel}}$ versus both reduced pressure $p^*\equiv p/(\bar{g}\,E^{*})$ (in red) and $p^*_{\text {r}}\equiv p/(\bar{g}_{\text {r}}\,E^{*})$ (in blue) for line contacts with various *H*, obtained with GFMD simulations. **b** The data points from (**a**) are used to calculate $\kappa ^{\text {1D}}$ and $\kappa ^{\text {1D}}_{\text {r}}$. (Color figure online)

Our numerical results of the relative contact area $a_{\text {rel}}$ versus both reduced pressure $p^*\equiv p/(\bar{g}\,E^{*})$ (in red) and $p^*_{\text {r}}\equiv p/(\bar{g}_{\text {r}}\,E^{*})$ (in blue) are shown in Fig. 2a for the three selected Hurst exponents. Notice that, the area-to-pressure relation appears linear for both $p^*$ and $p^*_{\text {r}}$; there is no dependence on Hurst exponent for $p^*_{\text {r}}$ and only negligible for $p^*$. However, if from the same data points the values of proportionality factors $\kappa ^{\text {1D}}\equiv a_{\text {rel}} / p^*$ and $\kappa ^{\text {1D}}_{\text {r}}\equiv a_{\text {rel}} / p^*_{\text {r}}$ are calculated, as presented in Fig. 2b, the following observations can be made: (1) The proportionality factor $\kappa ^{\text {1D}}$ is not a constant and varies rather significantly (on average by $\sim 25\%$ in the pressure range spanning from $p^* = 10^{-1}$ to $p^* = 10^{-3}$); (2) $\kappa ^{\text {1D}}$ depends significantly on *H*; (3) the proportionality factor $\kappa _{\text {r}}^{\text {1D}}$ is, on the contrary, constant and independent of *H*. We can therefore conclude that, similar to the case of single smooth asperities, also for line contacts the relation between relative contact area and reduced pressure is linear and independent of *H*, only if the RMSG is taken over the actual contact area. The value of the proportionality factor is $\kappa _{\text {r}}^{\text {1D}} \simeq 1.75$.

The results of $\kappa ^{\text {1D}}$ in Fig. 2b indicate also that one should be careful when fitting data for line contacts with laws that result in a constant and single valued $\kappa$. In his theory of contact, Persson [1, 29] demonstrated that the relative contact area may be approximated by $a_{\text {rel}} = \text {erf}(\sqrt{2} p^*)$. Later, Scaraggi et al. [10] proposed a correction to this equation so that it could be applied to line contacts:

11 $$\begin{aligned} a_{\text {rel}} = \text {erf}\left(\sqrt{2} \frac{p^*}{\varPsi (p^*)}\right). \end{aligned}$$

The correction function is defined as $\varPsi (p^*) = b_1 + (1-b_1)\; \text {erf}(b_2 \,p^*)$, where $b_1$ and $b_2$ are fitting parameters.

**Fig. 3:**
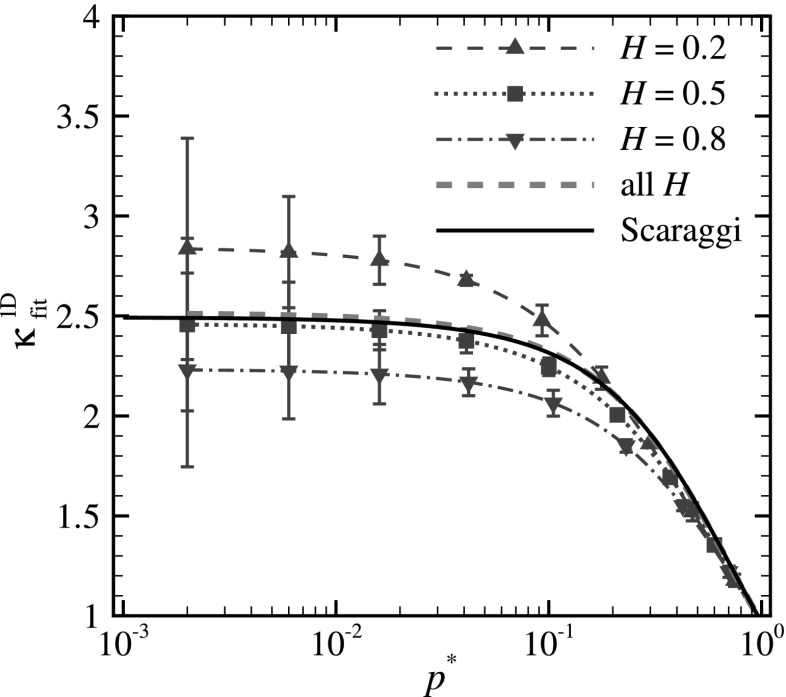
The proportionality factor $\kappa ^{\text {1D}}_\text {fit}$ versus reduced pressure $p^*$ for surfaces with Hurst exponent $H=0.2$, 0.5, and 0.8 are shown with red lines. The fit obtained for all *H* (dashed green line) is also included along with the fit calculated for the results obtained by Scaraggi et al. [10] (solid black line). All curves are obtained using the approximation of Eq. (11). (Color figure online)

If we apply the approximation of Eq. (11) and calculate one fit to all our numerical results of relative contact area $a_{\text {rel}}$ versus reduced pressure $p^*$, we obtain the proportionality factor $\kappa ^{\text {1D}}_\text {fit}$ presented with a dashed green line in Fig. 3. Our results are in good agreement with the results of boundary elements simulations performed by Scaraggi et al. [10] for profiles with various Hurst exponent and RMSG (solid black line). However, if we calculate independent fits on our numerical results of $a_{\text {rel}}$ versus $p^*$ for each value of the Hurst exponent (see the red curves in Fig. 3), we find that $\kappa ^{\text {1D}}_\text {fit}$ strongly depends on *H*, although for each Hurst exponent it is independent of reduced pressure when $p^* \lesssim 10^{-1}$. The latter is obviously expected, since linearity between relative contact area and reduced pressure is enforced by the fitting equation.

## Random Rough Surface Contacts

In this section, we will calculate the proportionality factors for random rough surface contacts, computing RMSG over nominal and actual contact area. Our aim is, first, to verify that $\kappa ^{\text {2D}}$ and $\kappa ^{\text {2D}}_{\text {r}}$ are in agreement and, second, to find the values of proportionality factors for surface contacts to be compared with the values obtained for line contacts in the previous section.

Here, we consider the same roughness parameters as in Sect. 3 except that $\varepsilon _\text {t} = 1/4$ and $\varepsilon _\text {f} = 1/64$ to keep the simulations computationally tractable with our facilities. Besides, the fractal dimension $D_\text {f}=3-H$ and in the power spectrum density of Eq. (10) the power of $q/q_{\text {r}}$ is replaced by $-2(1+H)$ [25].

**Fig. 4:**
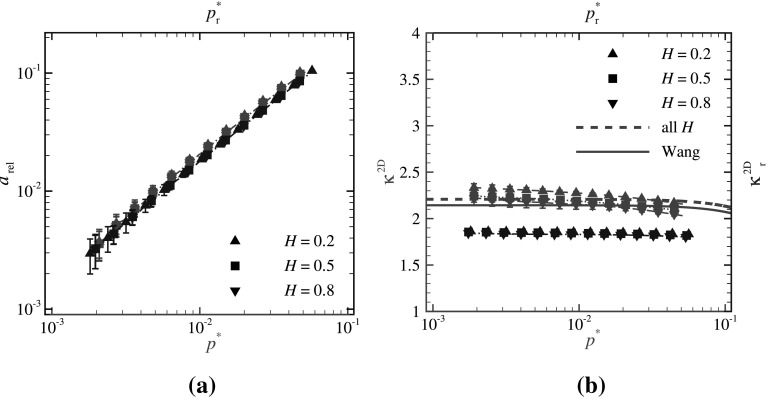
**a** GFMD predictions of the relative contact area $a_{\text {rel}}$ versus $p^*$ (red lines) and $p^*_{\text {r}}$ (blue lines) for three values of Hurst exponent $H=0.2$, 0.5, and 0.8. **b** The corresponding proportionality factors $\kappa ^{\text {2D}}$ and $\kappa ^{\text {2D}}_{\text {r}}$ are plotted against $p^*$ and $p^*_{\text {r}}$, respectively. Solid and dashed red lines are empirical fits to the results of Wang and Müser [30] and the current work, respectively. (Color figure online)

Figure 4a shows the results of relative contact area $a_{\text {rel}}$ versus both $p^*$ (in red) and $p^*_{\text {r}}$ (in blue). The data obtained for $p^*$ and for $p^*_{\text {r}}$ differ negligibly, i.e., much less than in the case of line contacts (compare with Fig. 2a). The corresponding proportionality factors $\kappa ^{\text {2D}}$ and $\kappa ^{\text {2D}}_{\text {r}}$ are shown in Fig. 4b.

In this figure, our results are compared with those obtained by Wang and Müser [30]. In their work, they assumed that the results are independent of the Hurst exponent and obtained an empirical fit analogous to [1] on the numerical results of Prodanov et al. [7]. We apply the same empirical fit to our numerical results (dashed red line in Fig. 4b). The difference between our curve and Wang’s is that the fit in [30] is obtained by using numerical results up to $p^* = 10^{1}$ while in our work only $p^*<10^{-1}$ is applied, as reaching higher values of $p^*$ demands computing power beyond our possibilities.

The results shown in Fig. 4b indicate that for surface contacts, the values of $\kappa ^{\text {2D}}$ and $\kappa ^{\text {2D}}_{\text {r}}$ (even without using an empirical fit) are negligibly dependent on the Hurst exponent and the reduced pressure. Moreover, compared to the 1D case (see Fig. 3), $\kappa ^{\text {2D}}$ and $\kappa ^{\text {2D}}_{\text {r}}$ differ less, as $\kappa ^{\text {2D}} \sim 2.20$ and $\kappa ^{\text {2D}}_{\text {r}} \simeq 1.88$.

## Comparison Between the Proportionality Factors for Line and Surface Contacts

**Fig. 5:**
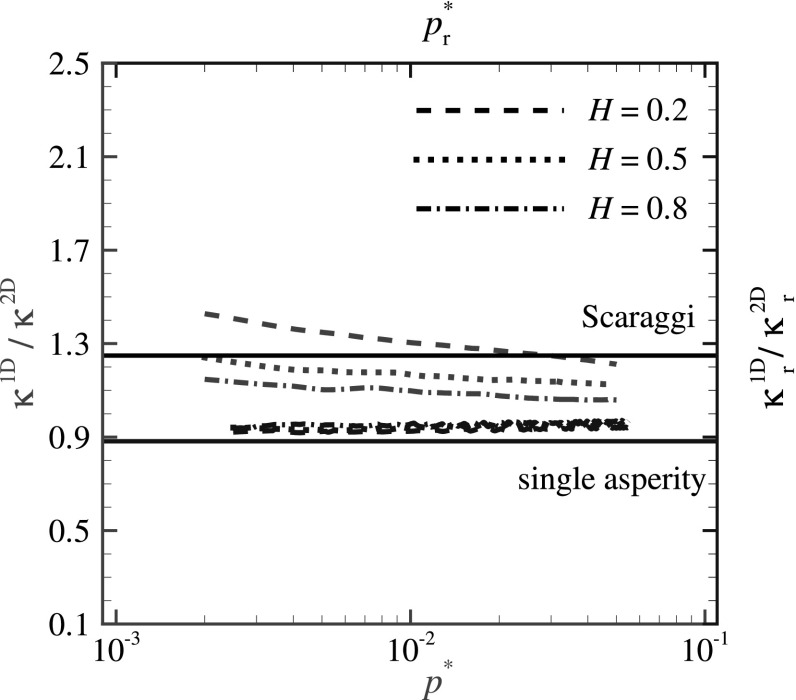
GFMD predictions for $\kappa ^{\text {1D}}/\kappa ^{\text {2D}}$ and $\kappa ^{\text {1D}}_{\text {r}}/\kappa ^{\text {2D}}_{\text {r}}$ versus reduced pressure values $p^*$ and $p^*_{\text {r}}$, respectively. Lines corresponding to the calculations of Scaraggi et al. [10] and the analytically obtained $\kappa ^{\text {1D}}_{\text {r}}/\kappa ^{\text {2D}}_{\text {r}}$ for single smooth asperity contacts (see Table 1) are also included in this figure

The results in terms of the ratio between the proportionality factors for line and surface contacts are shown in Fig. 5. Obviously, $\kappa ^{\text {1D}}/\kappa ^{\text {2D}}$ is not a constant but depends on both Hurst exponent *H* and reduced pressure $p^*$, similar to $\kappa ^{\text {1D}}$. The value of $\kappa ^{\text {1D}}_{\text {r}}/\kappa ^{\text {2D}}_{\text {r}}$ is constant and equal to 0.92.

Notice that when considering the RMSG over the nominal contact area, as for instance in the simulations by Scaraggi et al. [10], one finds $\kappa ^{\text {1D}}> \kappa ^{\text {2D}}$. Moreover, while $\kappa ^{\text {1D}}$ and $\kappa ^{\text {2D}}$ differ by $25 \%$, the difference between $\kappa _{\text {r}}$ for line and surface contacts is only $8\%$.

The calculated value for the cylinder and Hertzian contacts is $\kappa ^{\text {1D}}_{\text {r}}/\kappa ^{\text {2D}}_{\text {r}} \simeq 0.88$ (see Table 1) and is also presented in Fig. 5. This value is remarkably close to the value obtained for $\kappa ^{\text {1D}}_{\text {r}}/\kappa ^{\text {2D}}_{\text {r}}$ for random rough contacts. Therefore, we conclude that the 1D-to-2D scaling factor, $\kappa ^{\text {1D}}_{\text {r}}/\kappa ^{\text {2D}}_{\text {r}}\sim 0.9$, can be used for both random rough and Hertzian contacts.

## Analytical Derivation of $\kappa ^{\text {2D}}_{\text {r}}$ Using the Original GW Model

In the pioneering work of Greenwood and Williamson (GW) [21], an ensemble of identical spherical asperities was used to model the surface roughness. Here, we investigate whether by using ${\bar{g}}_{\text {r}}$ in the original GW model we can find a linear relation between the relative contact area $a_{\text {rel}}$ and the reduced pressure $p^{*}_{\text {r}}$.

Following [21], we assume that all asperity summits have radius *R* and the probability that an asperity has a height between *z* and $z + \text {d} z$ above the reference plane is $\phi (z) \, \text {d} z$. If the reference planes of the two surfaces are separated by distance *d*, then any asperity with height $z> d$ is in contact. These asperities are compressed on their centreline by $w = z - d$ and contribute by $\text {d} a_\text {act}$ and $\text {d} L$ to the total actual contact area ${a_\text {act}}$ and total load *L*, respectively. The values of ${\text {d} a_\text {act}}$ and $\text {d} L$ are given as

12 $$\begin{aligned} {\text {d} a_\text {act}} =&\, \pi {c}^2 = \pi R w, \end{aligned}$$

13 $$\begin{aligned} \text {d} L =&\frac{4 E^* w \sqrt{Rw} }{3}. \end{aligned}$$

Moreover,

14 $$\begin{aligned} a_{\text {rel}}\equiv&\int \limits _{d}^{\infty }{\text {d} a_\text {act}} \; \phi (z) \text {d}z, \end{aligned}$$

15 $$\begin{aligned} p\equiv&\int \limits _{d}^{\infty }\text {d} L \; \phi (z) \text {d}z. \end{aligned}$$

In the following, we consider two cases of asperity distribution: (i) Exponential: $\phi (z)=(1/\sigma )\exp (-|z|/\sigma )$ and (ii) Gaussian: $\phi (z) =(1/\sqrt{2 \pi \sigma ^2})\exp \left( -z^2/2\,\sigma ^2\right)$, where $\sigma$ is the root-mean-square height.

### Exponential Asperity Distribution

By substituting for ${\text {d} a_\text {act}}$, $\text {d} L$ and $\phi (z)$ in Eqs. (14) and (15), we obtain

16 $$\begin{aligned} a_{\text {rel}}=&\;\pi R \, \sigma \exp (-d/\sigma ), \end{aligned}$$

17 $$\begin{aligned} p=&\; \sigma E^* \sqrt{\pi R\,\sigma } \exp (-d/\sigma ). \end{aligned}$$

Furthermore, for a random rough surface contact

18 $$\begin{aligned} \bar{g}_{\text {r}}^2 = \frac{\int \limits _{d}^{\infty } \left[ \int \limits _{0}^{{c}} 2 \pi \rho \left( \frac{\partial h}{\partial \rho }\right) ^2 \text {d} \rho \right] \phi (z) \, \text {d} z}{a_{\text {rel}}}, \end{aligned}$$

where from Table 1 and [9] we have

19 $$\begin{aligned} \int \limits _{0}^{{c}} 2 \pi \rho \left( \frac{\partial h}{\partial \rho }\right) ^2 \text {d} \rho = \frac{\pi \, {c}^4}{2\,R^2}. \end{aligned}$$

Therefore,

20 $$\begin{aligned} \bar{g}_{\text {r}} = \sqrt{\frac{\sigma }{R}}. \end{aligned}$$

By substituting for the values of *p*, $a_{\text {rel}}$, and $\bar{g}_{\text {r}}$ in Eq. (3), the proportionality factor is obtained as

21 $$\begin{aligned} \kappa _{\text {r}}^{\text {2D}} = \sqrt{\pi }\simeq 1.77. \end{aligned}$$

Note that the obtained value of $\kappa _{\text {r}}^{\text {2D}}$ under the assumption of an exponential asperity distribution is independent of *R*, $\sigma$, and *d*.

### Gaussian Asperity Distribution

The same procedure shown above is performed to obtain $\kappa ^{\text {2D}}_{\text {r}}$. However, unlike the previous case, we reach a $\kappa _{\text {r}}^{\text {2D}}$ value which depends on $\sigma$ and *d*. Hence, we use the well-known asymptotic solution of BGT [31] for infinitesimal contacts, i.e., $(d/\sigma ) \rightarrow \infty$ and this again gives

22 $$\begin{aligned} \lim _{\frac{d}{\sigma }\rightarrow \infty } \kappa _{\text {r}}^{\text {2D}}\left(\tfrac{d}{\sigma }\right) = \sqrt{\pi }\simeq 1.77. \end{aligned}$$

Remarkably, the analytical value for $\kappa ^{\text {2D}}_{\text {r}}$ obtained by applying the original GW model, thus without considering elastic interactions, is in close agreement with our numerical result.

## Concluding Remarks

The relative contact area of rough surface contacts depends linearly on reduced pressure, with proportionality factor $\kappa$. It is customary to determine the reduced pressure considering the RMSG over the nominal contact area. However, we have here shown, with Green’s function molecular dynamics simulations, that $\kappa$ is not a constant in the case of line contacts, but depends rather strongly on Hurst exponent and reduced pressure.

Therefore, following the work of Müser [9] on axisymmetric asperities, we have calculated reduced pressure on line contacts by computing the RMSG over the actual contact area and reached the following conclusions:

- For line contacts, only when the RMSG is calculated over the *actual* contact area a linear relation exists between the relative contact area $a_{\text {rel}}$ and the reduced pressure $p^{*}_{\text {r}} \equiv p/(\bar{g}_{\text {r}}\,E^*)$, such that the proportionality factor $\kappa ^{\text {1D}}_{\text {r}}$ is independent of Hurst exponent and pressure. This holds true for rough contacts as well as for Hertzian asperities.
- A 1D-to-2D scaling factor is found for random rough and Hertzian contacts, i.e., $\kappa ^{\text {1D}}_{\text {r}}/\kappa ^{\text {2D}}_{\text {r}}\sim 0.9$.

Inspired by the results for line contacts, we have evaluated the RMSG over the actual contact area also in the framework of the original model by Greenwood and Williamson, and derived the analytical value for $\kappa ^{\text {2D}}_{\text {r}}$. Despite the fact that the model is simple and does not include elastic interactions, we found that the value of $\kappa ^{\text {2D}}_{\text {r}}$ is remarkably close to our GFMD numerical result for random rough surfaces.

Finally, it must be noted that measuring $\bar{g}$ experimentally is significantly easier than measuring $\bar{g}_{\text {r}}$ for which an in situ measurement of the actual contact area [8, 32] would be required, while for computer simulations there is no significant difference in effort.
